# Muscle Deoxygenation Rates and Reoxygenation Modeling During a Sprint Interval Training Exercise Performed Under Different Hypoxic Conditions

**DOI:** 10.3389/fphys.2022.864642

**Published:** 2022-07-15

**Authors:** Robert Solsona, Roméo Deriaz, Fabio Borrani, Anthony M. J. Sanchez

**Affiliations:** ^1^ Laboratoire Interdisciplinaire Performance Santé Environnement de Montagne (LIPSEM), Faculty of Sports Sciences, University of Perpignan Via Domitia (UPVD), Font-Romeu, France; ^2^ Institute of Sport Sciences, University of Lausanne, Lausanne, Switzerland

**Keywords:** blood flow restriction, occlusion, hypoxia, skeletal muscle, exercise training, altitude, gravity-induced BFR

## Abstract

This study compared the kinetics of muscle deoxygenation and reoxygenation during a sprint interval protocol performed under four modalities: blood flow restriction at 60% of the resting femoral artery occlusive pressure (BFR), gravity-induced BFR (G-BFR), simulated hypoxia (FiO_2_≈13%, HYP) and normoxia (NOR). Thirteen healthy men performed each session composed of five all-out 30-s efforts interspaced with 4 min of passive recovery. Total work during the exercises was 17 ± 3.4, 15.8 ± 2.9, 16.7 ± 3.4, and 18.0 ± 3.0 kJ for BFR, G-BFR, HYP and NOR, respectively. Muscle oxygenation was continuously measured with near-infrared spectroscopy. Tissue saturation index (TSI) was modelled with a linear function at the beginning of the sprint and reoxygenation during recovery with an exponential function. Results showed that both models were adjusted to the TSI (R^2^ = 0.98 and 0.95, respectively). Greater deoxygenation rates were observed in NOR compared to BFR (*p* = 0.028). No difference was found between the conditions for the deoxygenation rates relative to sprint total work (*p* > 0.05). Concerning reoxygenation, the amplitude of the exponential was not different among conditions (*p* > 0.05). The time delay of reoxygenation was longer in BFR compared to the other conditions (*p* < 0.05). A longer time constant was found for G-BFR compared to the other conditions (*p* < 0.05), and mean response time was longer for BFR and G-BFR. Finally, sprint performance was correlated with faster reoxygenation. Hence, deoxygenation rates were not different between the conditions when expressed relatively to total sprint work. Furthermore, BFR conditions impair reoxygenation: BFR delays and G-BFR slows down reoxygenation.

## Introduction

Sprint interval training (SIT) is a training method in vogue that was found to shortly improve both aerobic performance and repeated sprint ability (Burgomaster et al., 2006; Brocherie et al., 2017). SIT includes long sprints (e.g., 30 s) interspaced with long recovery periods (between 2 and 4 min) (Buchheit and Laursen, 2013). In the past few years, it has been observed that additional hypoxic stress during repeated sprint sessions amplifies several training adaptations, including muscle phosphocreatine content, repeated sprint ability, the onset of blood lactate accumulation, single sprint and Yo-Yo test performance (Millet et al., 2019).

Among the known hypoxic methods, there is systemic hypoxia, provoked by natural altitude or simulated altitude with hypoxic or hypobaric chambers (HYP), and local hypoxia, which can be obtained using cuffed blood flow restriction (BFR) and gravity-induced blood flow restriction (G-BFR) (Preobrazenski et al., 2020, 2021; Solsona et al., 2021). BFR consists in placing compression cuffs around the proximal part of the exercising muscles which induces ischemia and/or altered venous return according to the pressure exerted (Patterson et al., 2019). G-BFR is an easy-to-use method consisting in the inclination of an ergocycle to place the heart beneath the lower limbs, thus reducing blood availability to the working muscles (Preobrazenski et al., 2020, 2021; Solsona et al., 2021). However, the acute physiological effects of these methods during SIT sessions remain underexplored.

Muscle oxygenation kinetics have been studied during constant-intensity submaximal exercise with or without BFR (Salzmann et al., 2021). In this study, it was found that BFR accelerated the deoxygenation phases compared to normoxia (NOR), and increased total deoxygenation amplitude, especially during the last phase of exercise. Another study previously examined the muscle oxygenation kinetics during an SIT session performed in NOR (Buchheit et al., 2012). The authors found an association between sprint performance and muscle deoxygenation, as well as an increase of the deoxygenation and reoxygenation rates across the sprint repetitions. Specifically, the SIT session elicited near-to-maximal $\dot{V}O_{2}$ peak values, which were associated with a high degree of muscle deoxygenation. These data support the highly energetic demand of SIT protocols at the muscular level.

However, muscle deoxygenation and reoxygenation kinetics have never been studied and compared under different hypoxic conditions. This focus is of importance since muscle deoxygenation rates and the kinetics of muscle reoxygenation may be linked to performance and the replenishment of energy substrates. Indeed, previous studies showed differences in muscle oxygen availability and substrate utilization during sustained intermittent intense and continuous submaximal exercises (Christmass et al., 1999). Hence, the aim of this study was to compare the muscle deoxygenation rates and reoxygenation kinetics during an SIT exercise performed under different hypoxic protocols (BFR, G-BFR, HYP, and NOR) with a randomized crossover design. For this purpose, two models were used: a linear model for muscle deoxygenation at the onset of sprints and an exponential model during recovery. The hypotheses were that different deoxygenation rates would be observed according to the condition, with higher values in NOR due to more oxygen availability, compared to the other conditions, and according to the sprint number due to fatigue accumulation. Furthermore, muscle reoxygenation would be limited by the different hypoxic conditions.

## Methods

### Participants

The data have been collected during a previous experiment that aimed to examine the effects of SIT conducted in hypoxia or with BFR on mechanical, cardiorespiratory, and muscular O_2_ extraction responses (Solsona et al., 2021). Thirteen healthy young moderately trained men (mean ± standard deviation, age 24 ± 3 years; weight 73.8 ± 6.5 kg; height 179 ± 6 cm; body fat percentage 12.5 ± 2.1%; training frequency 8 ± 4 h per week) volunteered to participate in the study. They gave written consent to participate in the experiment, which was approved by the local ethics committee (VD-2021-00597). Participants completed the Physical Activity Readiness Questionnaire prior to the first session. All experiments were performed in accordance with the last Declaration of Helsinki. The participants were asked to maintain their dietary habits and to avoid alcohol consumption 48 h before each test. They did not take medication or dietary supplements during the studied period.

### Study Design

This crossover study aimed to compare the deoxygenation rates during an SIT session under different hypoxic conditions. Participants performed four SIT sessions under different conditions in a random order over 4 weeks. Sessions took place at least 5 days apart to avoid fatigue-related bias. Trials were performed at the same time of the day and within the same environmental conditions to minimize the effects of circadian cycles. Anthropometric measurements were carried out on the first visit and body fat percentage was estimated with the four skinfold method (Durnin and Womersley, 1974). Inflation cuffs (SC12D, cuff size 13 × 85 cm) and an inflation apparatus (E20/AG101 Rapid Cuff Inflation System, D. E Hokanson Inc., Bellevue, WA, United States) were used for BFR, which was only applied during the first 2 min of recovery at 60% of resting arterial occlusive pressure (AOP). Preliminary work determined these were the longest time and the highest pressure that could be tolerated by participants. Resting AOP was measured with an ultrasound linear probe (EchoWave II 3.4.4, Telemed Medical Systems, Milan, Italy) in a seated position on the right leg the day participants undertook the BFR condition. The pressure was progressively increased until no blood flow was detected in the femoral artery. A total of three measurements were taken with a one-minute recovery between each evaluation. The highest value recorded was retained and 60% of this value was used for the cuffs that were placed bilaterally during the exercise session. The cuffs were inflated immediately after the sprints for 2 min. Concerning G-BFR, a structure was built to allow participants to pedal in the supine position. The structure also permitted handgrip to mimic conventional cycling and to avoid body displacements during exercise. Mean vertical distance between the horizontal plane and the crank axis was about 35 cm. The laying position was adopted at the end of the warm-up and maintained until the end of the protocol. Regarding HYP, the inspired dioxygen fraction (FiO_2_) was set at 13.0% in a normobaric hypoxic chamber (ATS altitude training, Sydney, Australia). The warm-up was performed in HYP for this condition. NOR was performed below 400 m of altitude. The configuration (height and length) of both the saddle and the handlebars was recorded on the first visit to be reproduced in the subsequent tests. Participants had to maintain saddle contact and were verbally encouraged to provide maximal effort. Researchers did not provide verbal indications of time to avoid pacing strategies. Muscle oxygenation was continuously measured with a near-infrared spectroscopy (NIRS) probe (OxiplexTS, ISS, Champagne, United States). The device was calibrated before each session and was placed on the distal part of the vastus lateralis (VL) muscle. It was wrapped with an elastic band to limit extraneous light and movement.

### Sprint Interval Training Exercise

Exercise sessions consisted of a standardized warm-up of 16 min including 10 min at 100 W and 85 rpm, followed by two six-second sprints preceded by 54 s of resting. Thereafter, 4 min of passive recovery were allowed before the beginning of the SIT session, which comprised five all-out efforts of 30 s with 4 min of passive recovery. The session lasted until the recovery of the fifth sprint. The sessions took place in a controlled indoor environment with an ergocycle (Lode Excalibur Sport 911905, Lode B.V., Groningen, Netherlands) set on constant torque (0.8 Nm·kg^−1^). A three-second countdown indicated the beginning of the sprint, which was performed with a standing start (i.e., without inertia).

### Data Treatment

The acquisition frequency was 50 Hz and data were averaged every second. Tissue saturation index (TSI) was calculated as follows by the NIRS (Barstow, 2019):

$$TSI=\frac{\left[O_{2}Hb\right]}{\left[tHb\right]}\times 100$$

[O_2_Hb] and [tHb] represent the concentration of oxygenated haemoglobin and total haemoglobin concentrations, respectively. Then, data were treated with a low-pass second-order Butterworth filter with a cutoff frequency of 0.1 Hz. The slopes (a) and TSI_0_ (intercepts) of the TSI during the first 9 s of each sprint were calculated. This allowed to obtain a modeled TSI (mTSI) with a linear function (Bae et al., 2000):

$${mTSI}_{\left(t\right)}=a\times t+TSI_{0}$$

With t being the time (in seconds) elapsed from the start of the sprint. The slope represents the rate of deoxygenation per second (Buchheit et al., 2012). TSI_0_ is the intercept, corresponding with the TSI at the beginning of the sprint. The equation allowed the calculation of the coefficient of determination (R^2^) between the mTSI and the TSI data. The first 10 consecutive points (i.e., 9 s) were selected. Finally, the relative rate of deoxygenation (a_adj_) according to the sprint total work (W) was calculated as follows (Buchheit et al., 2012):

$$a_{adj}=\frac{a}{W}$$

**FIGURE 1:**
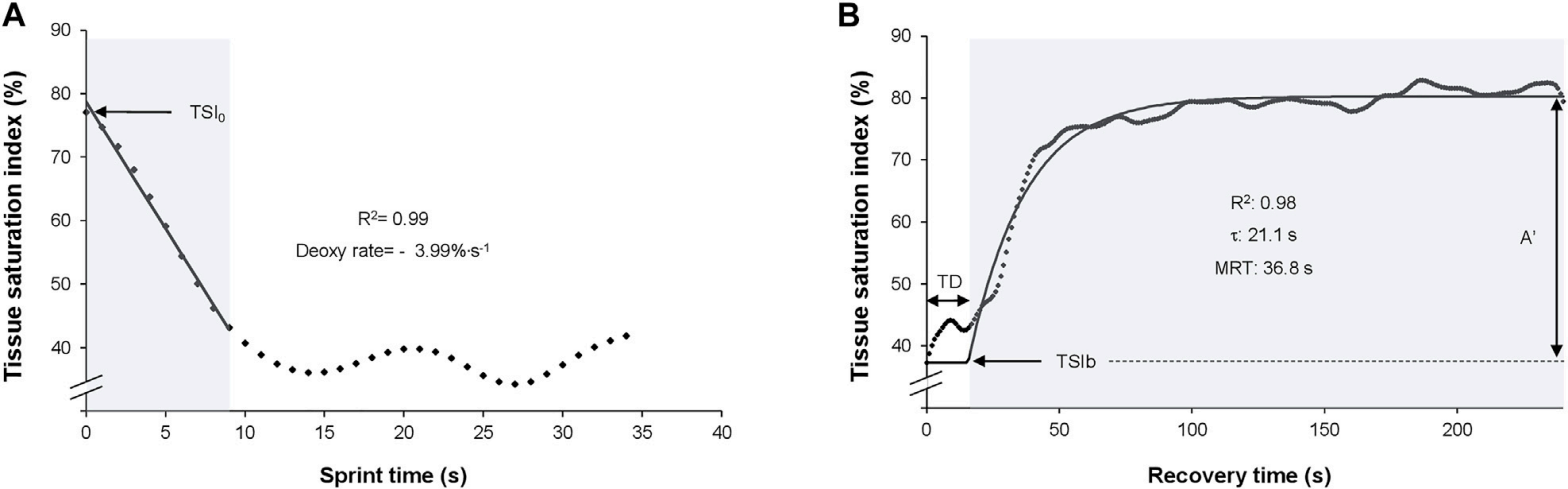
Measured (rhombuses) and modelled (line) tissue saturation index during a 30 s sprint **(A)** and during recovery **(B)** for a representative subject. Grey zone: modelled sections; TSI_0_: Tissue saturation index at the beginning of the sprint; R2: determination coefficient between measured and modelled data; Deoxy rate: deoxygenation rate per second (i.e., model slope). TSIb, TSI at the beginning of recovery; A', amplitude of the exponential; TD, time delay from the beginning of recovery until the beginning of the exponential; τ, time constant of the exponential; MRT, mean response time of the exponential. Of note, the values are representative and correspond to a subject’s illustrative sprint.

Of note, TSI is a better indicator of muscle oxygenation than deoxygenated haemoglobin when blood flow is not constant (Wolf et al., 2007). Figure 1 represents the measured and modelled TSI during a representative 30-second sprint.

ΔTSI is the absolute difference between maximal sprint TSI and minimal sprint TSI.

Muscle reoxygenation kinetics (TSIk) was modelled with an exponential function:

$${TSIk}_{\left(t\right)}=TSIb\;+\;A\left(1-e^{\frac{t-TD}{\tau }}\right)$$

With TSIb corresponding to the TSI at the beginning of recovery, A being total reoxygenation amplitude of the first exponential, TD is the time delay, τ is the exponential time constant and mean response time (MRT) equals to TD + τ. A′ is the amplitude of reoxygenation from the beginning of the exponential curve until the end of recovery (Tend), which was calculated as follows:

$$A^{\prime }=A\left(1-e^{\frac{-Tend-TD}{\tau }}\right)$$

The parameters of the model for deoxygenation (a, TSI_0_) and reoxygenation (A′, TD, τ, MRT) were determined with an iterative procedure by minimizing the sum of the mean squares of the differences between the estimated mTSI and TSIk models and the measured TSI. Power decrease percentage was calculated as follows (Glaister et al., 2008):

$$Power\;decrease\;percentage=100\times \frac{Sum\;of\;mean\;power\;of\;all\;the\;sprints}{Best\;mean\;power\times number\;of\;sprints}-100$$

### Statistical Analysis

The statistical analyses were performed using Jamovi (Version 1.6.23). Figures were designed with Microsoft Excel and PowerPoint. Linear mixed models were used to compare the conditions and the sprints number. The significance level was set at 0.05. Post hoc analyses were performed using pairwise comparisons with Holm’s correction. Effect sizes (d) are provided (trivial effect 0.10 < d < 0.20, small effect 0.20 < d < 0.50, medium effect 0.50 < d < 0.80 and large effect d > 0.80) (Cohen, 1992). Spearman correlations were performed between deoxygenation rates, reoxygenation kinetics and the other variables.

Furthermore, the coefficient of determination (R^2^) and the adjusted coefficient of determination (adjR^2^) were calculated from the beginning of the exponential until Tend:

$$\text{adj}R^{2}=1-\left(1-R^{2}\right)\frac{n-1}{n-k-1}$$

The coefficient k corresponds to the number of degrees of freedom of the model (i.e., three), n is the sample size.

## Results

No condition*sprint number interaction was detected for any variable.

### Performance

Peak power was lower in G-BFR compared to NOR (*p* < 0.01) and mean power was lower in G-BFR compared to the other conditions (*p* < 0.05). No difference between conditions was found (*p* > 0.05) in the performance decrease percentage (Table 1). Sprint exercises mean total work was 17 ± 3.4, 15.8 ± 2.9, 16.7 ± 3.4 and 18.0 ± 3.0 kJ for BFR, G-BFR, HYP and NOR, respectively. Mean total work was lower in G-BFR compared to the other conditions (*p* < 0.05).

**TABLE 1:** Peak and mean power output during sprints under the different conditions.

	BFR	G-BFR	HYP	NOR
Peak power (W)	766.6 ± 174.2	717.8 ± 135.0	783.0 ± 149.6	835.4 ± 176.0^†^
Mean power (W)	551.9 ± 107.4^†^	533.8 ± 97.6	557.4 ± 82.8^†^	593.4 ± 98.6^†^
Power decrease (%)	−16.1 ± 8.5	−12.7 ± 7.3	−12.5 ± 5.2	−12.9 ± 7.2

^†^significantly different from G-BFR (*p* < 0.05). Data are shown as mean ± standard deviation.

BFR, blood flow restriction; G-BFR, gravity-induced blood flow restriction; HYP, hypoxia; NOR, normoxia. Power decrease, percentage decrease between the best sprint and the mean sprints power.

### Goodness of the Fit and Coefficient of Determination for Deoxygenation Rates and Reoxygenation Kinetics

A total of 210/260 (R^2^>0.80) sprint exercises were modelled with the linear function and 148/260 (R^2^>0.80) sprint exercises were modelled with the exponential function. Indeed, an R > 0.90 (i.e., R^2^>0.80) represents a strong relationship between two variables (Taylor, 1990). In the linear model of muscle deoxygenation, the mean R^2^ between TSI and mTSI was 0.98 ± 0.02 (Figure 1). The coefficient of determination, as well as the adjusted coefficient of determination of the exponential model were 0.95 ± 0.04 (Figure 1).

### Muscle Deoxygenation Rates

**FIGURE 2:**
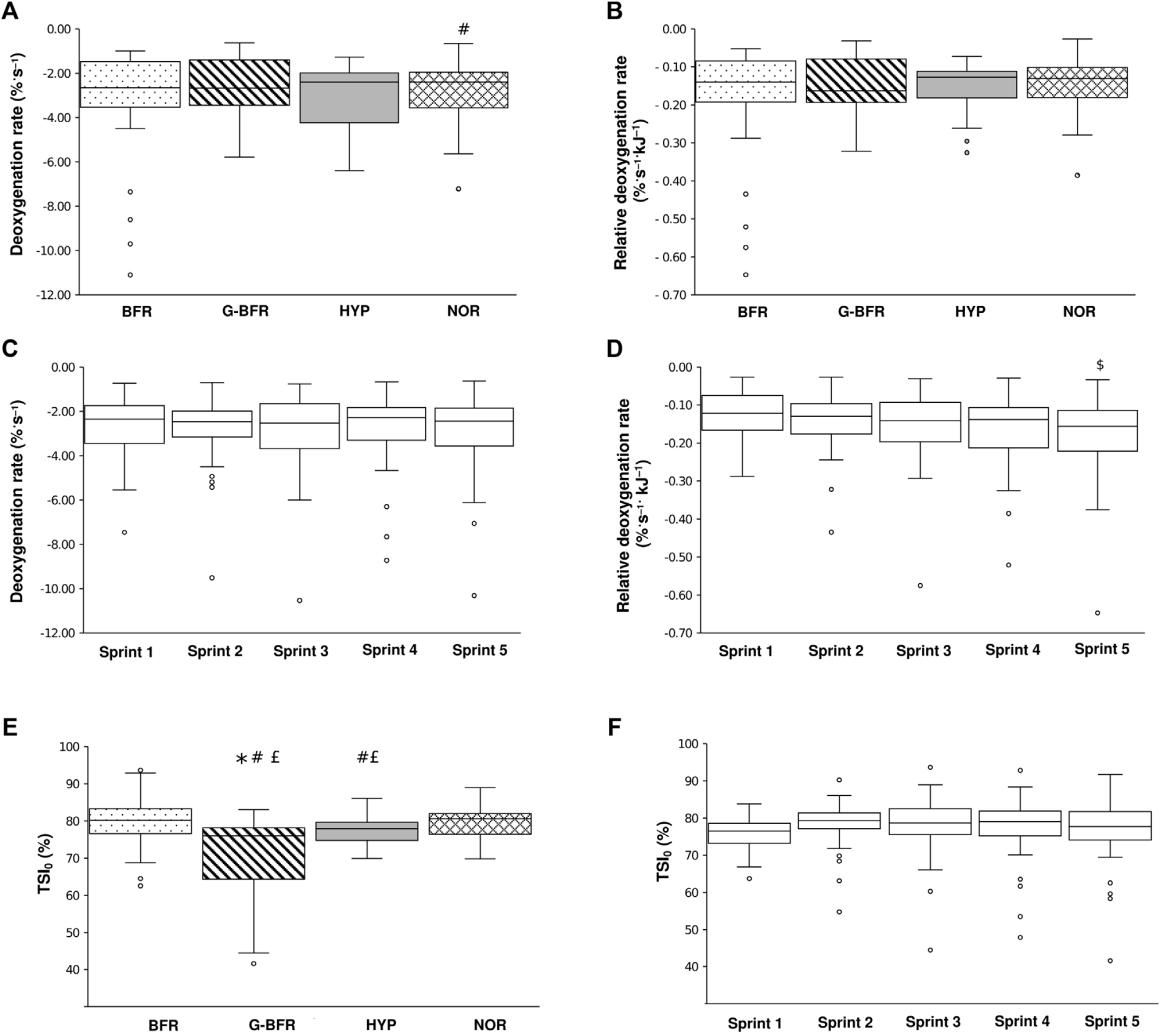
Deoxygenation rate and relative deoxygenation rate between conditions (**A** and **B** respectively) and between sprints (**C** and **D** respectively). Deoxygenation rates correspond to the slopes of the tissue saturation index (TSI)/time (in seconds) relationship. Relative deoxygenation rate considers total sprint work. Pre-sprint tissue saturation index (TSI_0_) comparison between conditions **(E)** and between sprints **(F)**. The dots represent outlier data points, the whiskers minimal and maximal values, the boxes the interquartile region, the horizontal line the median. Quartiles are also shown. £, significantly different from NOR; $, significantly different from sprint 1. *, significantly different from HYP; #, significantly different from BFR. Of note, condition graphs **(A**,**B**,**E)** include the five sprints averaged and sprint number graphs **(C**,**D**,**F)** include the four conditions averaged.

An effect of condition was detected for absolute deoxygenation rates (*p* = 0.014). According to post hoc tests, greater deoxygenation rates were observed in NOR compared to BFR (*p* = 0.028; d = 0.21, Figure 2A). The means were −2.4 ± 1.0% s^−1^ and −2.6 ± 1.2% s^−1^, respectively. However, when deoxygenation rates were expressed relatively to sprint total work, there was no significant difference (*p* > 0.05, Figure 2B): −0.15 ± 0.06% s^−1^ kJ for both BFR and NOR. Regarding sprint number, no difference on absolute deoxygenation rate was found (*p* > 0.05, Figure 2C). An effect of sprint number was found for adjusted deoxygenation rates (*p* = 0.030). Post hoc analyses showed that adjusted deoxygenation rates were higher in the fifth sprint compared to the first sprint (*p* = 0.041; d = 0.39, Figure 2D).

Importantly, significant differences were found regarding TSI_0_ between conditions: values were higher in BFR compared to G-BFR and HYP (*p* < 0.001; d = 1.11 and *p* = 0.014; d = 0.60, respectively, Figure 2E). TSI_0_ was also higher in HYP compared to G-BFR (*p* < 0.001; d = 0.77) and in NOR compared to G-BFR and HYP (*p* < 0.001; d = 0.99 and *p* = 0.035; d = 0.41, respectively, Figure 2E). Mean TSI_0_ values were 80.0 ± 4.4%, 72.4 ± 8.6%, 77.6 ± 3.7% and 79.2 ± 4.5% for BFR; G-BFR, HYP and NOR, respectively. Finally, regarding the sprint number, no difference was observed concerning TSI_0_ (*p* > 0.05, Figure 2F).

### Muscle Reoxygenation

The amplitude of the exponential (A’) did not present an effect of condition or sprint number (*p* > 0.05; Table 2). On the other hand, TD presented an effect of condition (*p* < 0.001). According to post hoc tests, TD was higher in BFR compared to G-BFR (*p* < 0.001; d = 0.96), HYP (*p* < 0.001; d = 1.12) and NOR (*p* < 0.001; d = 1.25). TSIb also showed an effect of condition (*p* < 0.001). Post hoc analyses revealed that TSIb was higher in BFR compared to G-BFR (*p* < 0.001; d = 1.35) and HYP (*p* = 0.001; d = 0.95). TSIb presented an effect of sprint (*p* = 0.017). According to post hoc analyses it was higher after sprints one and two compared to sprint five (*p* = 0.024; d = 0.65 and *p* = 0.011; d = 0.80, respectively). τ presented an effect of condition (*p* < 0.001), with higher values observed in G-BFR compared to BFR (*p* = 0.033; d = 0.24), HYP (*p* < 0.001; d = 0.76) and NOR (*p* < 0.001; d = 0.87). MRT was also different among the conditions (*p* < 0.001). MRT was higher in both BFR and G-BFR compared to HYP (*p* < 0.001; d = 1.39 and *p* < 0.001; d = 0.85, respectively) and NOR (*p* < 0.001; d = 1.62 and *p* < 0.001; d = 1.06, respectively).

**TABLE 2:** Parameters estimated for exponential curve fitting of individual reoxygenation response.

	BFR	G-BFR	HYP	NOR
A’ (%)	23.04 ± 7.12	29.34 ± 5.87	28.44 ± 9.98	27.05 ± 8.69
TD (s)	52.57 ± 40.20	24.20 ± 12.05^#^	18.54 ± 15.15^#^	14.71 ± 14.45^#^
τ (s)	50.12 ± 33.82^†^	60.82 ± 53.38	30.87 ± 16.77^†^	26.68 ± 15.77^†^
MRT (s)	102.69 ± 50.48	85.02 ± 55.55	49.41 ± 19.90^#†^	41.38 ± 17.68^#†^

^#^significantly different from BFR (*p* < 0.05).

^†^significantly different from G-BFR (*p* < 0.05). Data are shown as mean ± standard deviation.

BFR, blood flow restriction; G-BFR, gravity-induced blood flow restriction; HYP, hypoxia; NOR, normoxia. A’, amplitude of the exponential of the modelled TSI; TD, time delay of the exponential; τ, time constant of the exponential; MRT, mean response time of the exponential.

### Correlations

Negative correlations with deoxygenation rates were found for mean power (r = −0.16; *p* = 0.025), peak power (r = −0.26; *p* < 0.001), TSI_0_ (r = −0.31; *p* < 0.001) and ΔTSI (r = −0.78; *p* < 0.001). TSI_0_ was positively correlated to ΔTSI (r = 0.30; *p* < 0.001). Furthermore, mean power was negatively correlated with τ (r = −0.39; *p* < 0.001) and with MRT (r = −0.33; *p* < 0.001). Finally, peak power was positively associated with A’ (r = 0.28; *p* < 0.001), and negatively correlated to τ (r = −0.31; *p* < 0.001), TSIb (r = −0.22; *p* = 0.008) and MRT (r = −0.28; *p* < 0.001).

## Discussion

This crossover study aimed to model and to compare the muscle deoxygenation rates and reoxygenation kinetics during an SIT exercise performed under different hypoxic conditions in moderately trained men. An R^2^ of 0.98 was obtained between the measured and modelled TSI during sprints, which means that deoxygenation at the first phase of the sprint was linear. In addition, the exponential model fitted satisfactorily reoxygenation during the recovery periods. The present data show that, even if greater deoxygenation rates were observed in NOR compared to BFR, this difference disappeared when the deoxygenation rates were adjusted with sprint total work. Moreover, BFR delays and G-BFR slows down reoxygenation kinetics.

According to the data, absolute deoxygenation rates were not different between the sprints. However, greater adjusted deoxygenation rates were found in the fifth sprint compared to the first sprint, unlike the results of Buchheit and colleagues, who found a significant decrease in the adjusted deoxygenation rates with the repetition of sprints (Buchheit et al., 2012). However, it has been shown that glycogenolysis decreased since the second 30-s sprint (Bogdanis et al., 1996). It is also known that energy production from substrates depends less on phosphocreatine with the repetition of sprints (Parolin et al., 1999). Altogether, muscle energetic metabolism relies more on oxygen as long intermittent high intensity bouts are repeated. Of note, muscle contraction efficiency decreases with fatigue (Barclay, 1996), which would explain the increased deoxygenation rates for a given mechanical work during the last sprint. Furthermore, TSI_0_ was different between the conditions except between NOR and BFR. Specifically, HYP reduced TSI_0_, and G-BFR decreased it further. This result means that HYP, but mostly G-BFR, decreased basal TSI, but without any effect on the deoxygenation rates. Thus, while adding different hypoxic stress to an SIT session does not impact the muscular deoxygenation rate, it does affect pre-sprint TSI. Interestingly, this result suggests that basal TSI does not have an impact on deoxygenation rates during the current protocol. Lastly, the current study showed that TSI_0_ was lower in G-BFR compared to the other conditions. This result is in agreement with previous work from our laboratory, which showed that mean session TSI was also lower in G-BFR compared to BFR, NOR and HYP (Solsona et al., 2021).

A recent study from our laboratory highlighted that BFR could accelerate the decrease of TSI during constant-load submaximal exercise (Salzmann et al., 2021). Accordingly, a study with a repeated sprint protocol (i.e., 10-s sprints) showed that continuous BFR increases the amplitude of deoxyhaemoglobin, suggesting that deoxygenation rates were higher in this condition (Willis et al., 2019). The present study showed the opposite result, at least in the absolute deoxygenation rate values, probably because of the intermittent application of BFR. It could be speculated that deoxygenation rates would have been higher in BFR if it was applied during the sprints. On the other hand, as TSI represents the ratio of oxygenated haemoglobin to total haemoglobin, it is not possible to rule out an alteration of oxygen-rich blood delivery and/or an increase of total haemoglobin concentrations. Yet, both have recently been shown to increase proportionally during the present protocol under the BFR condition (Solsona et al., 2021), which would not affect the ratio and thus lead to an unmodified TSI.

The amplitude of reoxygenation was not limited by the different hypoxic conditions. Hence, 4 min of recovery are sufficient to reoxygenate muscles after SIT exercises under these conditions. Importantly, BFR delayed recovery compared to the other conditions (higher TD). On the other hand, τ was higher in G-BFR compared to BFR, NOR and HYP, meaning that the exponential curve increased slower, and that muscle reoxygenation is delayed by gravity. Indeed, we previously showed that mean session TSI was lower under this condition (Solsona et al., 2021). Moreover, TSIb was higher in BFR compared to HYP and G-BFR, which agrees with the higher minimal TSI value observed during the sprints (Solsona et al., 2021). Furthermore, MRT was longer under BFR and G-BFR compared to NOR and HYP. Blood flow restriction methods delay post-sprint recovery differently: while TD increased in BFR, τ increased in G-BFR. These results mean that BFR delays the beginning of the exponential and G-BFR slows down reoxygenation.

Deoxygenation rates were inversely correlated to peak power output, mean power, TSI_0_ and ΔTSI. This result means that performance is associated with higher deoxygenation rates, lower TSI_0_ and higher deoxygenation amplitude. On the other hand, TSI_0_ was correlated to ΔTSI, which suggests that higher pre-sprint TSI permits higher deoxygenation amplitudes. Finally, several reoxygenation variables were correlated with performance. Specifically, the amplitude of reoxygenation was correlated with peak power, meaning that higher levels of reoxygenation (because of lower post-sprint values) are useful for performance during SIT. Participants showing high power outputs also presented lower TSIb, reflecting their ability to further deoxygenate muscles during sprints. Finally, τ and MRT were shorter on participants who were able to achieve higher mean and peak power outputs. This result suggests that performance in the current protocol was associated with faster reoxygenation.

Some limitations must be acknowledged. Indeed, TSI is influenced by microvascular blood flow variation induced by thermoregulation (Grassi and Quaresima, 2016). Nevertheless, trials were performed in a laboratory environment with non-significant changes in temperature. In addition, oxygenation was measured in a small part of the VL muscle, making it difficult to extrapolate the results in the whole muscle. However, for this issue, a study demonstrated that deoxyhaemoglobin kinetics were similar between all the muscles of the quadriceps femoris during a moderate-intensity knee extension exercise (duManoir et al., 2010).

In conclusion, this study shows that adding different hypoxic stress to an SIT session did not have an impact on muscle deoxygenation rates in moderately trained athletes when total work was considered. Indeed, sprinting seems sufficient to cause high levels of deoxygenation rates, as it is observed in NOR. However, pre-sprint muscle oxygen availability was lower in HYP and G-BFR compared to both BFR and NOR, with a more pronounced effect observed for G-BFR compared to HYP. In addition, according to the exponential fitting, BFR delays and G-BFR slows down reoxygenation kinetics. Importantly, data also suggest that sprint performance is associated with faster reoxygenation and with higher amplitude of both deoxygenation and reoxygenation.

## Data Availability

The raw data supporting the conclusions of this article will be made available by the authors, without undue reservation.
