# Erratum: Muscle deoxygenation rates and reoxygenation modeling during a sprint interval training exercise performed under different hypoxic conditions

**DOI:** 10.3389/fphys.2023.1181484

**Published:** 2023-03-16

**Authors:** Frontiers Production Office

**Affiliations:** Frontiers Media SA, Lausanne, Switzerland

**Keywords:** blood flow restriction, occlusion, hypoxia, skeletal muscle, exercise training, altitude, gravity-induced BFR

An omission to the **Funding** section of the original article was made in error. The following sentence has been added: “Open access funding was provided by the University of Lausanne.”

The original version of this article has been updated.

